# Negative Immune Regulator TIPE2 Promotes M2 Macrophage Differentiation through the Activation of PI3K-AKT Signaling Pathway

**DOI:** 10.1371/journal.pone.0170666

**Published:** 2017-01-25

**Authors:** Ruiling Liu, Tingting Fan, Wenwen Geng, Youhai H. Chen, Qingguo Ruan, Cui Zhang

**Affiliations:** 1 GuangDong Pharmaceutical University, Guangzhou, People's Republic of China; 2 Center for Antibody Drug, Institute of Biomedicine and Biotechnology, Shenzhen Institutes of Advanced Technology, Chinese Academy of Sciences, Shenzhen, People's Republic of China; 3 Department of Pathology and Laboratory of Medicine, University of Pennsylvania School of Medicine, Philadelphia, Pennsylvania, United States of America; Queen Mary University of London, UNITED KINGDOM

## Abstract

Macrophages play important roles in the regulation of the innate and adaptive immune responses. Classically activated macrophages and alternatively activated macrophages are the two major forms of macrophages and have opposing functionalities. Tumor necrosis factor-α-induced protein 8–2 is expressed primarily by immune cells and negatively regulates type 1 innate and adaptive immune responses to maintain immune tolerance. While previous studies indicate that TIPE2 promotes M2 but inhibits M1 macrophage differentiation, the underlying molecular mechanism by which TIPE2 promotes M2 macrophage differentiation remains unclear. Our current study shows that TIPE2-deficient bone-marrow cells are defective in IL-4 induced M2 macrophage differentiation in vitro. Mechanistic studies revealed that TIPE2 promotes phosphoinositide metabolism and the activation of the down-stream AKT signaling pathway, which in turn leads to the expression of markers specific for M2 macrophages. In addition, our results showed that Tipe2-deficiency does not affect the activation of the JAK-STAT6 signaling pathway that also plays an important role during M2 macrophage differentiation. Taken together, these results indicate that TIPE2 promotes M2 macrophage differentiation through the activation of PI3K-AKT signaling pathway, and may play an important role during the resolution of inflammation, parasite control, as well as tissue repair.

## Introduction

Macrophages play important roles in the regulation of the immune response and are involved in health and disease [1, 2]. The main function of macrophages is to respond to pathogens and regulate the immune response through antigen presentation and cytokine production [3, 4]. Depending on the micro-environmental stimuli, macrophages can differentiate into classically activated macrophages (M1) and alternatively activated macrophages (M2) [5]. Th1-related cytokines like IFN-γ/TNF-α, endogenous stress signals and exogenous stimuli such as LPS (lipopolysaccharides) and dsDNA will polarize macrophages towards an M1 phenotype. In contrast, Th2-related cytokines like IL-4/IL-13 and immunomodulatory cytokines IL-10/TGF-β will polarize macrophages towards an M2 phenotype [6, 7]. In addition, it has been reported that glucocorticoid hormones, apoptotic cells and immune complexes can also induce macrophages to an M2-like phenotype [8, 9]. M1 macrophages can secrete inflammatory cytokines such as IL-1β, TNF-α, IL-12, IL-18 and IL-23 [10–12]. They have also been shown to up-regulate the expression of the intracellular protein suppressor of cytokine signaling 3 (SOCS3) [13, 14] and promote the production of NO from L-arginine through the activation of inducible nitric oxide synthase (iNOS) [15, 16]. Although M1 macrophages play important roles in the promotion of Th1 responses and mediate resistance to pathogens, they have also been implicated in initiating and sustaining inflammation, and therefore can also be detrimental to health [17, 18]. M2 macrophages are able to secrete high amounts of immunomodulatory cytokines such as IL-10 and TGF-β and convert arginine metabolism to express ornithine and polyamine [19]. M2 macrophages possess anti-inflammatory functions and are able to promote tissue remodeling and repair, dampen inflammation, help in the clearance of parasites and enhance tumor progression [20–22].

Tumor necrosis factor-α induced protein-8-like 2 (TNFAIP8L2 or TIPE2) belongs to TNFAIP8 family and was identified to be over-expressed in mice with EAE (Experimental Autoimmune Encephalomyelitis) [23, 24]. Accumulating evidence suggests that TIPE2 is a negative regulator of innate and adaptive immune response [23]. TIPE2 is preferentially expressed in lymphoid tissues and Tipe2-deficient cells are hyper-responsive to Toll-like receptor (TLR) and T cell receptor (TCR) activation [23]. A previous study showed that TIPE2 is able to limit phagocytosis and oxidative burst in macrophages by binding to and blocking Rac GTPases [25]. Additionally, TIPE2 negatively regulates inflammation by switching arginine metabolism from nitric oxide synthase to arginase in macrophages [26]. Mechanistic studies revealed that TIPE2 may inhibit the activation of NF-κB and the phosphorylation of JNK and p38 following LPS challenge [23, 27], suggesting that TIPE2 may inhibit M1 macrophage differentiation. Recently Xu et al found that TIPE2 alleviates experimental Systemic lupus erythematosus (SLE) through induction of macrophage polarization to a M2 phenotype [28]. However, the underlying molecular mechanism that TIPE2 promotes M2 macrophage differentiation remains unclear.

In the current study, we investigated the molecular mechanism that TIPE2 promotes M2 macrophage differentiation using Tipe2-deficient mice. Our study demonstrates that TIPE2 promotes M2 macrophage differentiation through the activation of Phosphoinositide 3-kinase (PI3K)-AKT signaling pathway.

## Materials and Methods

### 1. Animals

8 to 11-week-old wild-type and *Tipe2*^*-/-*^ mice in the C57BL/6 background were used in the experiments and kept under pathogen-free conditions at the animal core facility of the Shenzhen Institutes of Advanced Technology, Chinese Academy of Sciences. All efforts were made to minimize the number of mice used and to prevent animal distress, pain, and injury. Carbon dioxide (CO2) was used for euthanasia of mice. All procedures were preapproved by the Animal Care and Use Committee of Shenzhen Institutes of Advanced Technology, Chinese Academy of Sciences (Permit Number: SIAT-IRB-130306-A0000).

### 2. Preparation of bone marrow-derived macrophages

Bone marrow-derived macrophages (BMM) were generated from femoral and tibial bone marrow cells as previously described [25]. Briefly, 3x10^6^/mL bone marrow precursors from WT and *Tipe2*^*-/-*^ mice were seeded in complete RPMI1640 culture medium supplemented with 20% L929 culture supernatant in 10cm plates. Half of the medium was replaced with fresh medium on days 4. On day 7, macrophages were about 95% F480+ as determined by flow cytometry and ready for further experiments.

### 3. RNA isolation and real-time quantitative PCR

Total RNA was isolated using TRIzol reagent according to the manufacturer’s instructions (Life Technologies). RNA samples were reversely transcribed with prime script Rtreagent kit (Takara). Real-time quantitative PCR analysis was performed using specific primers for mouse *Fabp4* (F, 5'- GCTTGTCTCCAGTGAAAACTTC-3'; R, 5'-GTCGTCTGCGGTGATTTCATC-3'),*Ym1*(F, 5'-AGAAGGGAGTTTCAAACCT-3'; R, 5'-ATCTGACGGTTCTGAGGAG-3'), *Atgl*, *Mgl-1*, *Mgl-2*, *Arg-1*, *iNOS*, *Fizz-1* and *Cd36* [29] in an Applied Biosystems 7500 system using THUNDERBIRD SYBR qPCR Mix (TOYOBO). Relative levels of gene expression were determined using GAPDH [30] as the control.

### 4. Cytokine assay

For cytokine assays, BMM were cultured in medium without L929 culture supernatant for 24 hours. Cells were then either untreated (M0), treated with IFN-γ (50ng/ml, PeproTech) plus LPS (10 ng/mL, eBioscience) (M1) or IL-4 (10ng/ml, PeproTech) (M2). Culture supernatants were collected 24 h (for IL-12) and 48 h (for IL-10) later; Cytokine concentration was determined by quantitative enzyme-linked immunosorbent assay (ELISA) per manufacturer’s recommendations (ebioscience).

### 5. Urea and NO detection

The samples were collected as described above. NO levels in culture supernatant and urea levels in total protein extracts were determined with the use of commercial kits (from BioAssay Systems for urea detection and from Beyotime Biotechnology for NO detection, respectively) according to the manufacturer’s instructions.

### 6. Intracellular staining

For intracellular staining, BMM were cultured in medium without L929 culture supernatant for 24 hours. Cells were then either untreated or treated with IL-4 (10ng/ml, PeproTech) for 20 min. After wash twice with PBS+2%FBS, cells were fixed with 4% formaldehyde for 30 min, permeabilized with perm buffer (Biolegend) for 10 min and incubated for 60 min with a 1:100 dilution of Phospho-AKT (T308) antibody (Cell Signaling Technology), Phospho-PDK1 (Ser241) antibody (Cell Signaling Technology) or Phospho-STAT6 (Tyr641) antibody (Millipore). Cells were washed three times and incubated for 30 min in 1:1000 diluted DyLight™ 488 Donkey anti-rabbit IgG antibody (Biolegend) in perm buffer. Stained cells were then washed and analyzed on CytoFLEX flow cytometry system (Beckman Coulter, Inc). Data were analyzed with the FlowJo software.

### 7. Immunoblotting

Protein extracts from macrophages were spotted onto a PVDF membrane. The membrane was then blocked using 5% nonfat milk in TBS-T, and incubated with 1:200 diluted rabbit anti-phosphatidylinositol 4,5-bisphosphate (PIP2) (Echelon), rabbit anti-Phosphatidylinositol (3,4,5)-trisphosphate (PIP3) (Echelon) or 1:4000 diluted mouse anti-ß-actin (Sigma) for 60 min at room temperature. After wash with TBS-T, the membrane was incubated with the secondary HRP-conjugated anti-rabbit IgM (1:2000, for PIP2 and PIP3, Abcam) or anti-mouse IgG (1:4000, for ß-actin, Santa Cruz) for 30 min at room temperature. The cells were then washed with TBS-T and signals were detected by chemiluminescence (Thermo scientific) and quantified by densitometry using ImageJ software. Relative levels of expression were determined using β-actin as the control.

### 8. Statistical analysis

The significance of the differences of the level of cytokines, urea and NO, proteins, lipids, and mRNA was determined by Student t test.

## Results

### 1. TIPE2 promotes M2 but inhibits M1 macrophage differentiation

**Fig 1:**
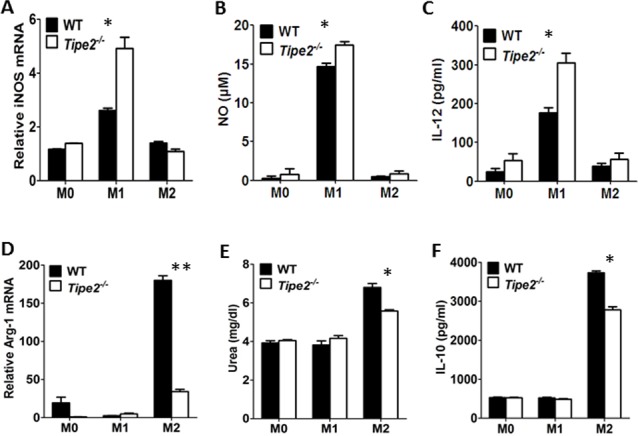
TIPE2 promotes M2 but inhibits M1 macrophage differentiation. Bone marrow derived macrophages from WT and Tipe2-deficient mice (n = 3) were untreated (M0), treated with IFN-γ (50 ng/ml) and LPS (10 ng/ml) (M1) or IL-4 (10 ng/ml) (M2) for 24 h. **(A, B & C)** To detect the expression of markers specific for M1 macrophages, mRNA level of iNOS was examined by real-time RT-PCR **(A)**. The production of NO **(B)** and IL-12 **(C)** in the culture supernatant were examined by a commercial NO detection kit and ELISA, respectively. **(D, E & F)** To detect the expression of markers specific for M2 macrophages, mRNA level of Arg-1 was examined by real-time RT-PCR **(D)**. The amount of urea in the protein extract was examined by a commercial urea detection kit **(E)** and the production of IL-10 in the culture supernatant was examined by ELISA **(F)**. For A and D, data shown are fold increase over the group with the lowest expression level. For all figures, data shown are mean±SD of one representative experiment. The experiments were repeated three times with similar results. * p<0.05, ** p<0.01.

In order to explore the role of TIPE2 in macrophage differentiation, bone marrow derived macrophages (BMM) from wild-type and Tipe2-deficient mice were treated with either IFN-γ plus LPS to induce M1 macrophages or IL-4 to induce M2 macrophages. Our results revealed that under M1 macrophage-inducing condition, TIPE2-deficient macrophages produced more iNOS mRNA (Fig 1A). Consistent with this result, the NO production in the culture supernatant of Tipe2-deficient macrophages was significantly increased (Fig 1B). IL-12 is an important inflammatory cytokine secreted by M1 macrophages. Our results showed that Tipe2-deficient macrophages produced more IL-12 than WT macrophages under M1 macrophage-inducing condition (Fig 1C). However, when macrophages were treated with M2 macrophage-inducing condition, Tipe2-deficient macrophages produced less Arg-1 mRNA (Fig 1D) and urea (Fig 1E). In addition, we found that the production of IL-10, an important cytokine secreted by M2 macrophages, was significantly decreased by Tipe2-deficient macrophages (Fig 1F). Taken together, these results demonstrate that TIPE2 promotes M2 but inhibits M1 macrophage differentiation in vitro.

### 2. TIPE2 promotes AKT but does not affect STAT6 signaling pathway

**Fig 2:**
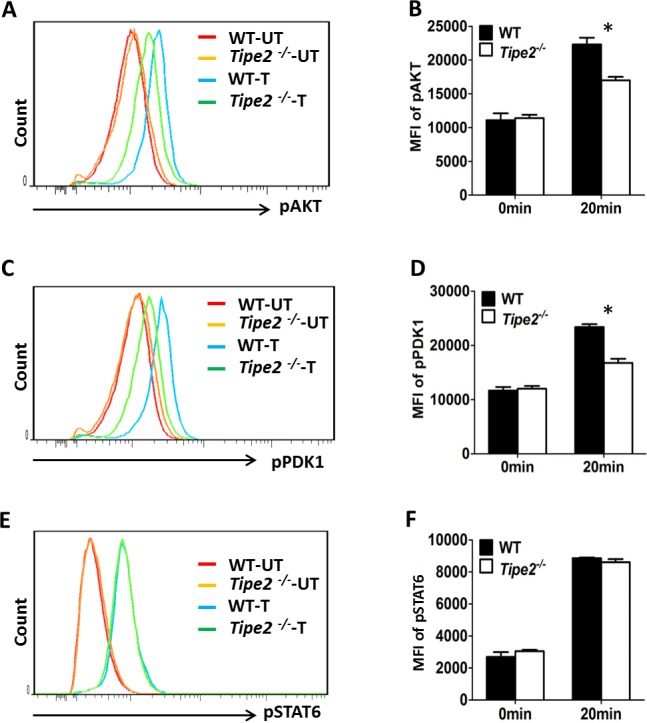
Activation of PI3K-AKT but not JAK-STAT6 signaling pathway is defective in Tipe2-deficient macrophages. Bone marrow derived macrophages from WT and Tipe2-deficient mice (n = 3) were either untreated (UT) or treated with IL-4 (10 ng/ml) (T) for 20 min. **(A)** Cells were stained with anti-Phospho-AKT (T308) and analyzed by flow cytometry. **(B)** The MFI (mean fluorescence intensity) of phosphorylated AKT (pAKT) was determined using FlowJo software. **(C)** Cells were stained with anti-Phospho-PDK1 (Ser241) and analyzed by flow cytometry. **(D)** The MFI of phosphorylated PDK1 (pPDK1) was determined using the same method as shown in (B). **(E)** Cells were stained with anti-Phospho-STAT6 (Tyr641) and analyzed by flow cytometry. **(F)** The MFI of phosphorylated STAT6 (pSTAT6) was determined using the same method as shown in (B). Results are representative of three independent experiments. For B, D and F, Data shown are mean±SD of one representative experiment. * p<0.05.

Because both AKT and STAT6 signaling pathways are important for M2 macrophage differentiation, next we examined whether TIPE2 promotes M2 macrophage differentiation by regulating these two signaling pathways. Our results showed that Tipe2-deficient macrophages had a significant decrease in AKT (Fig 2A and 2B) phosphorylation after treatment with IL-4. We also found that the phosphorylation of Phosphoinositide-dependent kinase-1 (PDK1), a molecule that directly phosphorylates AKT at T308, was also decreased (Fig 2C and 2D). In contrast, STAT6 phosphorylation was comparable between WT and Tipe2-deficient macrophages (Fig 2E and 2F). These results indicate that TIPE2 may promote the differentiation of M2 macrophages by enhancing the phosphorylation of PDK1 and AKT.

**Fig 3:**
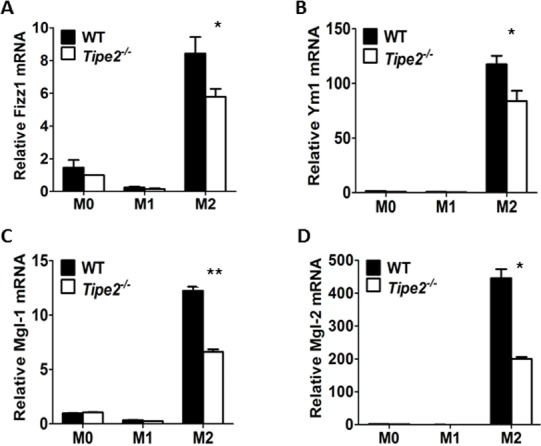
Expression levels of M2 macrophage markers regulated by PI3K-AKT signaling pathway are significantly decreased by Tipe2-deficient macrophages. Bone marrow derived macrophages from WT and Tipe2-deficient mice (n = 3) were untreated (M0), treated with IFN-γ (50 ng/ml) and LPS (10 ng/ml) (M1) or IL-4 (10 ng/ml) (M2) for 24 h. Expression levels of Fizz1 **(A)**, Ym1 **(B)**, Mgl-1 **(C)** and Mgl-2 **(D)** were determined by real time RT-PCR and normalized to the expression level of GAPDH. Data shown are mean±SD of one representative experiment. The experiments were repeated three times with similar results. * p<0.05, ** p<0.01.

**Fig 4:**
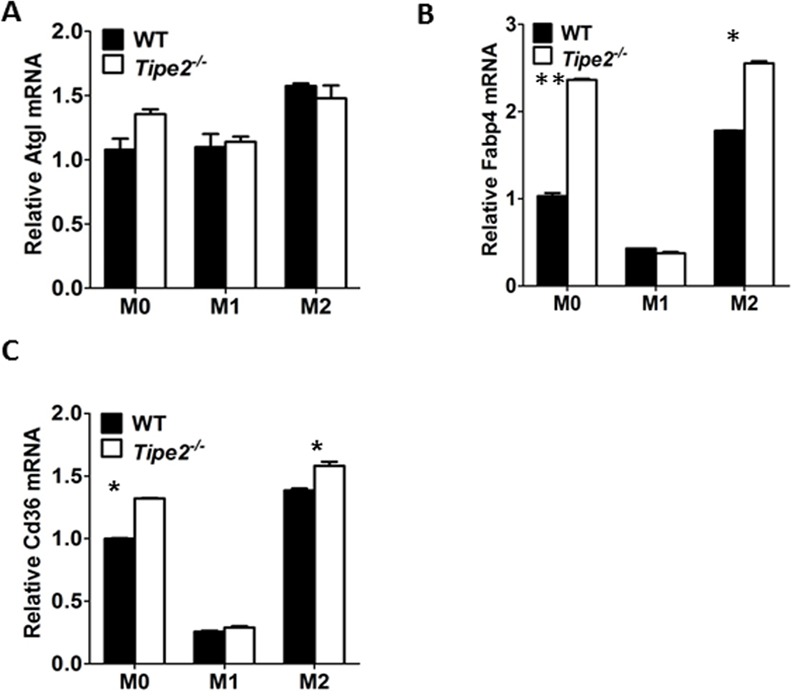
Expression levels of M2 macrophage markers regulated by JAK-STAT6 signaling pathway are either increased or not affected by Tipe2-deficient macrophages. Bo Bone marrow derived macrophages from WT and Tipe2-deficient mice (n = 3) were untreated (M0), treated with IFN-γ (50 ng/ml) and LPS (10 ng/ml) (M1) or IL-4 (10 ng/ml) (M2) for 24 h. Expression levels of Atgl **(A),** Fabp4 **(B)** and Cd36 **(C)** were determined by RT-PCR and normalized to the expression level of GAPDH. Data shown are mean±SD of one representative experiment. The experiments were repeated three times with similar results. * p<0.05.

To further confirm that TIPE2 promotes the activation of AKT signaling pathway but does not affect STAT6 signaling pathway, we examined the expression of key M2 macrophage markers that were regulated by these two signaling pathways, respectively. Our results showed that expression levels of key M2 markers regulated by AKT signaling pathway such as *Arg1*, *Fizz1*, *Mgl1*, *Mgl2* and *Ym1* were significantly reduced by Tipe2-deficient macrophages under M2 macrophage differentiation condition (Fig 3). However, expression levels of key M2 markers regulated by STAT6 signaling pathway such as *Fabp4*, *Cd36* and *Atgl* were either not affected or increased by Tipe2-deficient macrophages (Fig 4). It is worth noting that expression of Arg1 and Fizz1 could be regulated by both signaling pathways. Taken together, these results are consistent with our finding that TIPE2 promotes the activation of AKT but does not affect STAT6 signaling pathway.

### 3. TIPE2 promotes phosphoinositide metabolism

**Fig 5:**
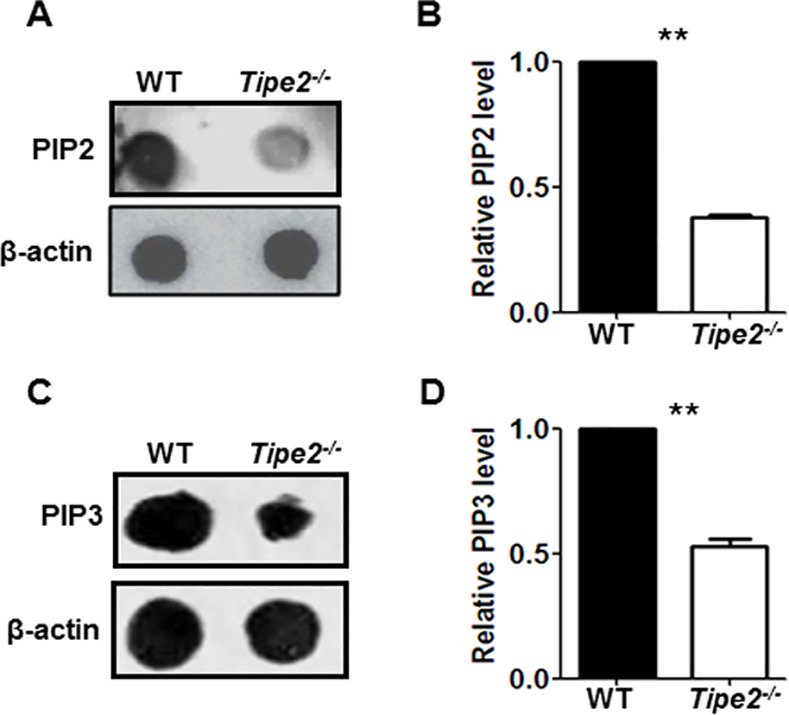
Cellular levels of PIP2 and PIP3 were significantly decreased by Tipe2-deficient macrophages. Bone marrow derived macrophages from WT and Tipe2-deficient mice (n = 3) were treated with IL-4 (10 ng/ml) for 20 min. **(A)** Cellular level of PIP2 was estimated by dot blot with anti-PIP2 antibody. **(B)** Relative expression level of PIP2 was determined using β-actin as the control and quantified by densitometry using ImageJ software. **(C)** Cellular level of PIP3 was estimated by dot blot with anti-PIP3 antibody. **(D)** Relative expression level of PIP3 was determined using same method as shown in (B). For A and C, results are representative of three independent experiments. For B and D, data shown are mean±SD for cells from three independent experiments. ** p<0.01.

**Fig 6:**
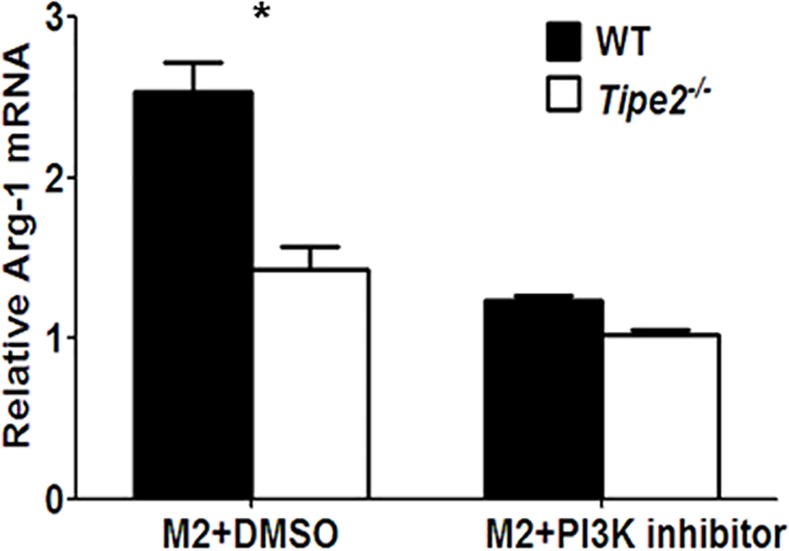
Inhibition of PI3K reduces the difference of Arg-1 expression between WT and Tipe2-deficient M2 macrophages. Bone marrow derived macrophages from WT and Tipe2-deficient mice (n = 3) were treated with or without PI3K inhibitor NSC23766 (10 μM) for 30 min before stimulated with IL-4 (10 ng/ml) for 24 h. Expression level of Arg-1 was determined by real time RT-PCR and normalized to the expression level of GAPDH. Data shown are mean±SD of one representative experiment. The experiments were repeated three times with similar results. * p<0.05.

Although we have shown that TIPE2 promotes M2 macrophage differentiation by enhancing the phosphorylation of PDK1 and AKT, how TIPE2 regulates this process remains unclear. Because both PDK1 and AKT are downstream molecules of second messenger PIP3, which was generated through PI3K activation, we next examined whether phosphoinositide metabolism was affected by TIPE2 deficiency. Our results showed that cellular levels of PIP2 (Fig 5A and 5B) and PIP3 (Fig 5C and 5D) were significantly decreased by Tipe2-deficient macrophages after IL-4 treatment. In addition, we found that PI3K blockade suppressed M2 macrophage differentiation and reduced the difference of Arg-1 expression between WT and Tipe2-deficient M2 macrophages (Fig 6). These results indicate that TIPE2 promotes M2 macrophage differentiation through the activation of PI3K-AKT signaling pathway. Interestingly, while cellular levels of PIP2 and PIP3 were still significantly decreased by Tipe2-deficient macrophages under M1 macrophage-inducing condition (S1 Fig), the phosphorylation of AKT is comparable between WT and Tipe2-deficient cells (S2 Fig).

TIPE3 is another TIPE protein family member. It has been reported that TIPE3 is essential for the activation of the PI3K-AKT pathway. This prompted us to examine whether TIPE3 also contributes positively to M2 macrophage polarization. However, we found that expression level of TIPE3 in M0, M1 and M2 macrophages is extremely low compared to that in intestine (S3 Fig). This result is consistent with published data that TIPE3 expression is barely detectable in macrophage cell line RAW264.7 [31].

## Discussion

Previously it has been shown that TIPE2 is a negative regulator of type 1 innate and adaptive immune response [23]. Following LPS challenge, TIPE2 negatively regulates inflammation by switching arginine metabolism from nitric oxide synthase to arginase in macrophages [26]. These results indicate that TIPE2 may play an important role during macrophage differentiation. Depending on the microenvironment stimuli, macrophages can differentiate into M1 or M2 macrophages in vivo [32]. In vitro, M1 macrophages can also be induced by IFN-γ and LPS while M2 macrophages can be induced by Th2 cytokines such as IL-4 and IL-13 [33]. Those in vitro induced macrophages have been widely used to study the function of macrophages since they share the same phenotype and function as in vivo polarized macrophages. Our results showed that TIPE2 inhibits IFN-γ and LPS induced M1 macrophage differentiation in vitro. This could be due to the inhibition of NF-κB and MAPK signaling pathway by TIPE2 based on published data [23]. Furthermore, we found that Tipe2-deficient bone-marrow cells are defective in IL-4-induced M2 macrophage differentiation in vitro, which is consistent with a recently published paper showing that TIPE2 overexpression induced macrophage polarization to a M2 phenotype [28].

However, the underlying molecular mechanism that TIPE2 promotes M2 macrophage differentiation remains unclear. Stimulation of macrophages with IL-4 leads to the activation of the transcription factor STAT6, which is important for M2 macrophage polarization [34–36]. In addition, PI3K-AKT signaling pathway is activated by the IL-4 treatment in parallel to the JAK-STAT6 pathway and attenuated AKT signaling underlies aberrant M2 macrophage polarization [29, 37, 38]. Recently it has been shown that the PI3K signaling pathway could suppress M1 macrophage differentiation and enhances the M2 phenotype through its downstream molecule miR-21 [39]. Thus, TIPE2 could directly regulate molecules that are involved in M2 macrophage differentiation. Alternatively, because M1 macrophage activation may reciprocally inhibit M2 macrophage differentiation, TIPE2 could promote M2 macrophage differentiation indirectly through the inhibition of M1 macrophage differentiation.

In order to investigate whether TIPE2 regulates M2 macrophage differentiation directly, we examined the activation of both PI3K and JAK-STAT6 signaling pathways, which have been shown before to be important for M2 macrophage differentiation. Our study revealed that TIPE2 promotes the activation of PI3K-AKT but does not affect JAK-STAT6 signaling pathway. The PI3K family of proteins plays an important role in cell proliferation and differentiation, apoptosis, and glucose transportation [40–43]. Second messenger PIP3, which is generated through PI3K activation in the plasma membrane, can bind PH-domain containing proteins such as AKT and PDK1 and promote the phosphorylation of AKT directly or through PDK1 [44]. Previous study has revealed that all TIPE family proteins including TIPE2 contain a highly conserved TIPE2 homology (TH) domain capable of binding phosphoinositides [31]. Furthermore, TIPE3, another TIPE protein family member, binds to phosphoinositides through its TH domain, which is essential for the activation of the PI3K-AKT pathway [31]. These results prompted us to test the hypothesis that TIPE2 may promote the activation of the PI3K-AKT pathway through enhancing PIP3 signaling. Our study showed that TIPE2 is indeed required for PI3K-mediated phosphoinositide metabolism. However, it is not known how TIPE2 promotes this process. We hypothesize that TIPE2 may regulate the distribution of lipid second messengers in response to stimuli. Alternatively, TIPE2 may also function as a lipid-presenting protein to enhance the activity of PI3K. Further studies are needed to elucidate the mechanisms that promote phosphoinositide metabolism by TIPE2.

Both TIPE2 and TIPE3 can bind phosphoinositide and promote the activation of the PI3K-AKT pathway. However, unlike TIPE3, TIPE2 can also interact with Caspase 8, Rac1 and Rgl/RalGDS and negatively regulates their function [23, 25, 45, 46]. Therefore, TIPE2 appears to be able to function as both a negative and a positive regulator depending on the cell types and conditions. Activation of AKT can be mediated by both Ral GTPases (non-canonical activation) and PI3K (canonical activation). Generation of PIP3 by PI3K at the membrane is essential for canonical AKT activation [47]. By contrast, the non-canonical AKT activation requires the activation of PDK1 by RalGDS, and this in turn leads to increased phosphorylation of AKT [48]. We hypothesize that, while activation of AKT can be mediated by both RalGDS and PI3K in M1 macrophage, activation of AKT was mainly mediated by PI3K in M2 macrophage. Because it has been reported that TIPE2 inhibits Ral-induced activation of AKT [46], TIPE2 appears to be able to function as both a negative and a positive regulator of AKT activation in M1 macrophage.

## Conclusion

TIPE family member TIPE2 can promote the activation of PI3K-AKT signaling pathway, which leads to enhanced M2 macrophage differentiation. Thus, the importance of TIPE2 in controlling type 1 inflammation may result from its role in switching macrophage differentiation from classically activated M1 macrophages to alternatively activated M2 macrophages.

## Supporting Information

S1 Fig Cellular levels of PIP2 and PIP3 were significantly decreased by Tipe2-deficient macrophages under M1 macrophage-inducing condition. (PDF)

S2 Fig Phosphorylation of AKT is comparable between WT and Tipe2-deficient M1 macrophage. (PDF)

S3 Fig TIPE3 expression is barely detectable in macrophages. (PDF)

## Abbreviations

TIPE2: Tumor necrosis factor-α induced protein-8-like 2
PI3K: Phosphoinositide 3-kinase
JAK: Janus kinase
STAT6: Signal transducers and activators of transcription 6
BMM: Bone marrow derived macrophage
iNOS: Inducible nitric oxide synthase
PIP2: Phosphatidylinositol 4,5-bisphosphate
PIP3: Phosphatidylinositol (3,4,5)-trisphosphate
PDK1: Phosphoinositide-dependent kinase-1
MAPK: Mitogen-activated protein kinase
