# Supplementary material for: Negative Immune Regulator TIPE2 Promotes M2 Macrophage Differentiation through the Activation of PI3K-AKT Signaling Pathway
Source: PLoS One. 2017 Jan 25;12(1):e0170666. doi: 10.1371/journal.pone.0170666 (PMC5266285; doi:10.1371/journal.pone.0170666)
Supplement: S1 Fig — (PDF) [file pone.0170666.s001.pdf]

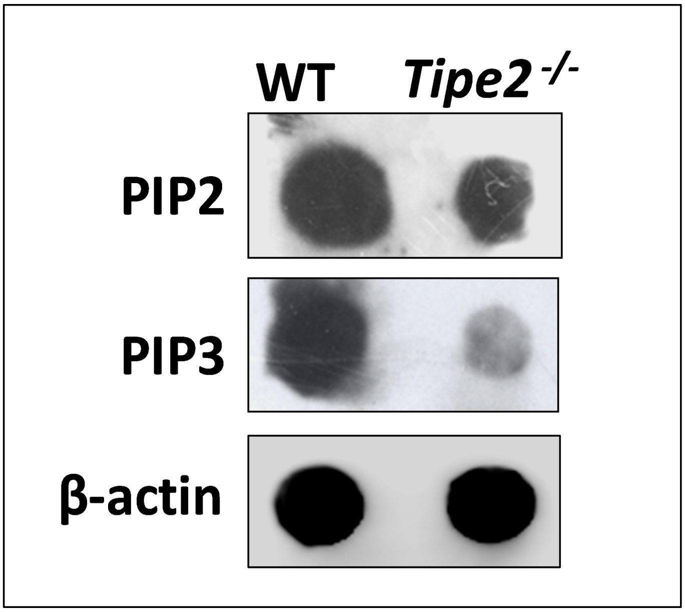

S1 Fig . Cellular levels of PIP2 and PIP3 were significantly decreased by Tipe2-deficient macrophages under M1 macrophage-inducing condition. Bone marrow derived macrophages from WT and Tipe2-deficient mice (n=3) were treated with IFN-γ (50 ng/ml) and LPS (10 ng/ml) for 20 min. Cellular levels of PIP2 and PIP3 were estimated by dot blot with anti-PIP2 or anti-PIP3 antibody, respectively. Result is representative of three independent experiments.
