# Supplementary material for: Negative Immune Regulator TIPE2 Promotes M2 Macrophage Differentiation through the Activation of PI3K-AKT Signaling Pathway
Source: PLoS One. 2017 Jan 25;12(1):e0170666. doi: 10.1371/journal.pone.0170666 (PMC5266285; doi:10.1371/journal.pone.0170666)
Supplement: S2 Fig — (PDF) [file pone.0170666.s002.pdf]

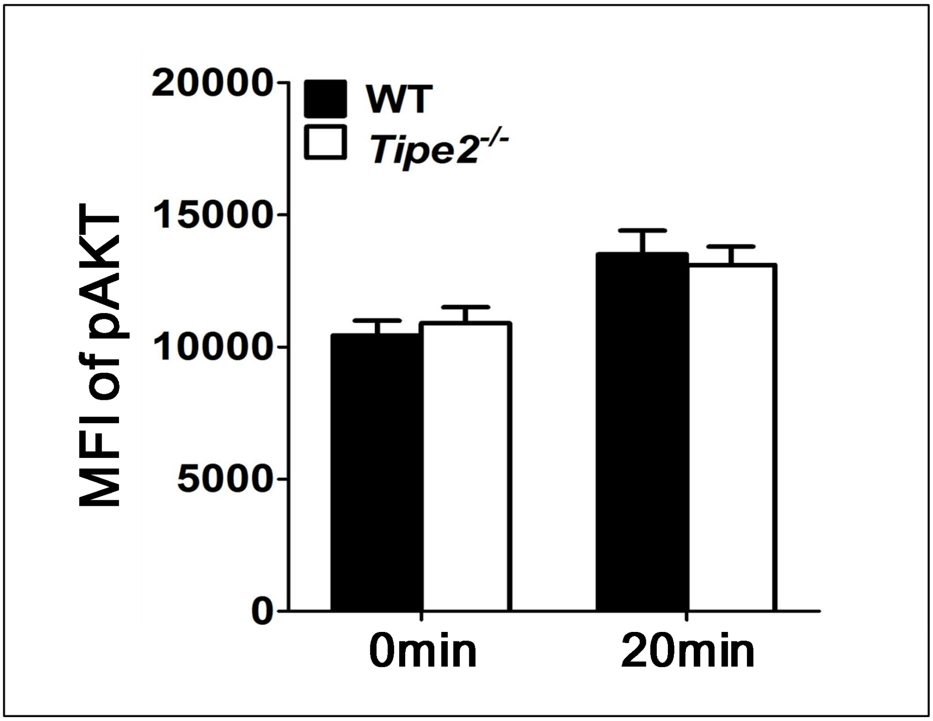

S2 Fig. Phosphorylation of AKT is comparable between WT and Tipe2-deficient M1 macrophage. Bone marrow derived macrophages from WT and Tipe2-deficient mice (n=3) were either untreated (0 min) or treated with IFN-γ (50 ng/ml) and LPS (10 ng/ml) for 20 min (20 min). Cells were stained with anti-Phospho-AKT (T308) and analyzed by flow cytometry. The MFI (mean fluorescence intensity) of phosphorylated AKT (pAKT) was determined using FlowJo software. Result is representative of two independent experiments.
