# Supplementary material for: Negative Immune Regulator TIPE2 Promotes M2 Macrophage Differentiation through the Activation of PI3K-AKT Signaling Pathway
Source: PLoS One. 2017 Jan 25;12(1):e0170666. doi: 10.1371/journal.pone.0170666 (PMC5266285; doi:10.1371/journal.pone.0170666)
Supplement: S3 Fig — (PDF) [file pone.0170666.s003.pdf]

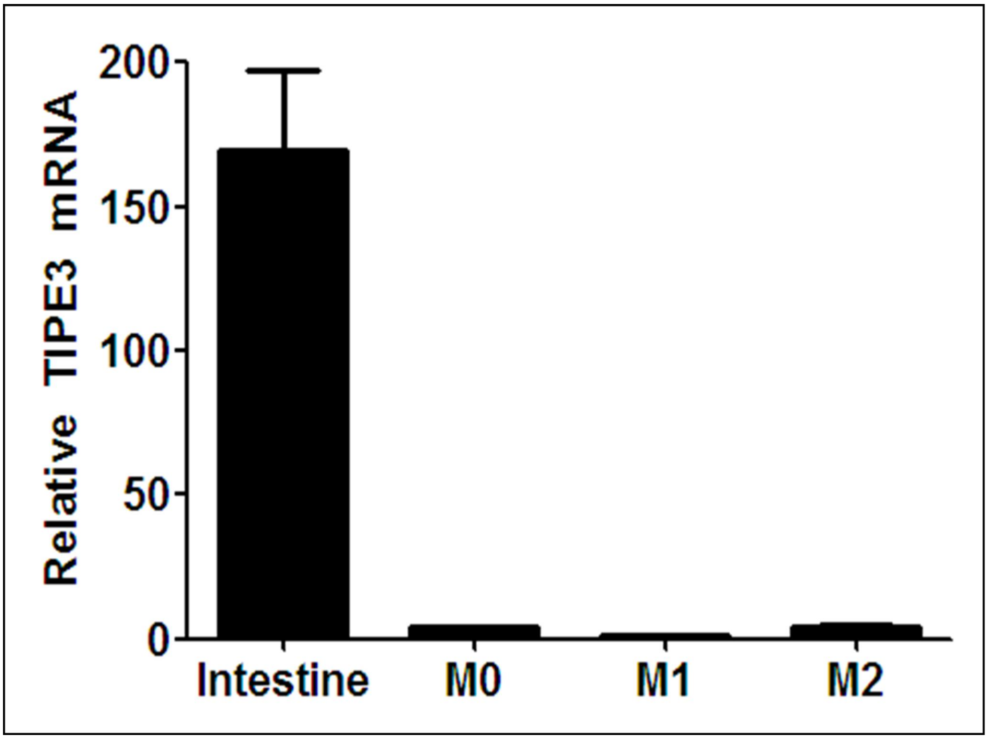

S3 Fig. TIPE3 expression is barely detectable in macrophages. Bone marrow derived macrophages from WT mice were untreated (M0), treated with IFN-γ (50 ng/ml) and LPS (10 ng/ml) (M1) or IL-4 (10 ng/ml) (M2) for 24 h. mRNA level of TIPE3 was examined by real-time RT-PCR. TIPE3 mRNA expression in intestine was used as the positive control. Result is representative of two independent experiments.
